# Bacterial Etiology and Antibiotic Resistance Profile of Community-Acquired Urinary Tract Infections in a Cameroonian City

**DOI:** 10.1155/2016/3240268

**Published:** 2016-09-07

**Authors:** Rolf Nyah-tuku Nzalie, Hortense Kamga Gonsu, Sinata Koulla-Shiro

**Affiliations:** ^1^Faculty of Medicine and Biomedical Sciences, Yaoundé, Cameroon; ^2^Microbiology Laboratory, Yaoundé University Hospital Centre, Yaoundé, Cameroon; ^3^Infectious Disease Unit, Yaoundé Central Hospital, Yaoundé, Cameroon; ^4^National Research Agency on AIDS and Viral Hepatitis, Yaoundé, Cameroon

## Abstract

*Introduction*. Community-acquired urinary tract infections (CAUTIs) are usually treated empirically. Geographical variations in etiologic agents and their antibiotic sensitivity patterns are common. Knowledge of antibiotic resistance trends is important for improving evidence-based recommendations for empirical treatment of UTIs. Our aim was to determine the major bacterial etiologies of CAUTIs and their antibiotic resistance patterns in a cosmopolitan area of Cameroon for comparison with prescription practices of local physicians.* Methods*. We performed a cross-sectional descriptive study at two main hospitals in Yaoundé, collecting a clean-catch mid-stream urine sample from 92 patients having a clinical diagnosis of UTI. The empirical antibiotherapy was noted, and identification of bacterial species was done on CLED agar; antibiotic susceptibility testing was performed using the Kirby-Bauer disc diffusion method.* Results*. A total of 55 patients had samples positive for a UTI. Ciprofloxacin and amoxicillin/clavulanic acid were the most empirically prescribed antibiotics (30.9% and 23.6%, resp.); bacterial isolates showed high prevalence of resistance to both compounds.* Escherichia coli* (50.9%) was the most common pathogen, followed by* Klebsiella pneumoniae* (16.4%). Prevalence of resistance for ciprofloxacin was higher compared to newer quinolones.* Conclusions*.* E. coli* and* K. pneumoniae* were the predominant bacterial etiologies; the prevalence of resistance to commonly prescribed antibiotics was high.

## 1. Introduction

Urinary tract infections (UTIs) are among the most common bacterial infections diagnosed in community health practice [1]. Community-acquired urinary tract infections are often treated empirically with broad-spectrum antibiotics because point-of-care bacterial testing is unavailable. Several studies [2, 3] show geographic variations in etiologic agents of UTIs and their resistance patterns to antibiotics.

Laboratory results of antimicrobial susceptibility testing with urinary tract infections are usually obtained two to three days after sampling; therefore, in the majority of community-acquired UTI (CAUTI) cases, treatment choice is empirically based on the predictable spectrum of etiological microorganisms and available data reflecting antibiotic resistance of previous infections [3]. Considering that, as with many community-acquired infections, resistance rates to antimicrobials commonly used in treatment of UTI are increasing and susceptibility of microorganisms shows significant geographical variation [4], studies aimed at increasing knowledge of local etiologic agents of UTIs and their resistance patterns to antibiotics are necessary to guide clinicians in empiric treatment. Recent studies [5, 6] show a growing problem of antibiotic resistance in Cameroon, thereby establishing a need for continuous surveillance of antibiotic susceptibility of uropathogens.

The aim of this study was to determine the prevailing bacterial etiologic agents associated with community-acquired UTIs and their susceptibilities to commonly empirically prescribed antibiotics in cases of UTIs to generate data that will improve the efficacy of empiric treatment of this infection.

## 2. Methods

### 2.1. Setting

This study was carried out at the University Hospital Centre and the Central Hospital of Yaoundé, which are among the four main hospitals of the city. About ten minutes apart by road, they receive most of the patients in Yaoundé. The University Hospital Centre has a microbiology laboratory for the analysis of patient samples and the training of microbiologists.

### 2.2. Study Design and Patients

This was a cross-sectional descriptive study conducted from January to April 2014. The study population was composed of patients with UTI symptoms presenting at the outpatient units and patients who developed these symptoms within 48 hrs after hospitalization. We included patients who presented with UTI symptoms and whose urine samples showed significant bacterial growth (≥10^5^ CFU/mL) associated with a white blood cell count of >10^4^/mL. We excluded patients who underwent antibiotic treatment or had been hospitalized within the previous week or whose urine samples showed no significant bacterial growth or white blood cell counts.

### 2.3. Ethical Considerations

Ethical clearance was obtained from the Institutional Ethics Committee for Research of the FMBS of the University of Yaoundé I. Data were collected after patients signed a written informed consent. For minors, their parents or guardians signed on their behalf. Identification codes were used for data treatment to ensure confidentiality and privacy of information; results were used solely for scientific purposes.

### 2.4. Data, Sample Collection, and Analysis

During outpatient consultations and ward rounds, sociodemographic characteristics and presenting complaints were collected from consenting patients using a pretested, validated questionnaire. Also noted was the empirical antibiotherapy put in place by the consulting physician after clinical diagnosis of UTI was made.

The clean-catch midstream technique was used to collect urine samples of at least 20 mL into a sterile Dynarex container. For female patients, after proper positioning of the thigh, they were instructed to spread the labia and clean the area with soaped swabs, then pass a small amount of urine into the toilet, and finally urinate into the container. For male patients, after hand washing, a clean-catch midstream urine sample was collected after retraction of the prepuce (if present) and cleaning of the glans with soaped swabs.

Each sample container was labeled and transported (within one hr) in a nonrefrigerated cooler box to the microbiology laboratory of the Yaoundé University Hospital Centre for analysis.

Leukocyte counts were determined by microscopy using a Malassez slide, and the urine sample was cultured, using a standard loop calibrated to contain 0.01 mL of urine, onto CLED agar. Inoculated plates were incubated at 37°C aerobically for 18–24 hrs. After incubation, the plates were examined and growth characteristics noted. Samples were usually inoculated before cell content analysis to reduce the risk of sample contamination. Colonies were counted on the inoculated medium and multiplied by the loop volume to determine bacterial count. Bacteria were identified using standard biochemical procedures and Gram staining.

### 2.5. Antibiotic Susceptibility Testing

Antibiotic susceptibility was determined using the Kirby-Bauer disc diffusion method on Mueller-Hinton agar as described by the National Committee for Clinical Laboratory Standards (presently called Clinical and Laboratory Standards Institute [7]). The isolates were tested against commonly prescribed antibiotics: ampicillin, amoxicillin/clavulanic acid, nalidixic acid, cotrimoxazole, gentamicin, ofloxacin, ciprofloxacin, levofloxacin, nitrofurantoin, ceftriaxone, and fosfomycin.

Whenever the bacterium isolated was found to be resistant to the empirically prescribed antibiotic, the physician was consulted on whether the patient had to modify his/her treatment to achieve a cure.

### 2.6. Statistical Analysis

Data were entered and analysed using EPI INFO 7.0 and Microsoft Excel 2007. The Fisher exact test was used for comparison of proportions; *p* values less than 0.05 were considered statistically significant. Results were summarized as means and percentages and presented in the form of graphs and tables.

## 3. Results

A total of 92 patients who fulfilled our inclusion criteria were sampled. Of these, 55 patients had urine samples that showed significant bacterial growth, giving us a prevalence of 59.8%. Most (92.7%) of these patients were from the outpatient unit, while only 7.3% were hospitalized patients.

The age of our patients ranged from 15 to 75 years, with a mean of 39.2 ± 17.6 years and a median of 35 years. Of the 55 significant samples, 46 were from females while 9 were from males. Analysing prevalence with respect to gender, females (83.6%) had a higher prevalence of infection than males (16.4%). UTI prevalence was significantly related to gender (*p* value = 0.002).

**Figure 1:**
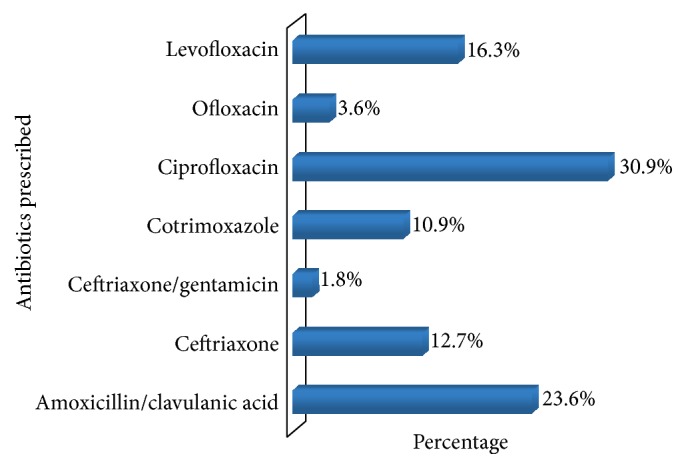
Relative amounts of antibiotics prescribed empirically for CAUTIs.

The most frequent empirically prescribed antibiotics with the patient sample were ciprofloxacin (30.9%) and amoxicillin/clavulanic acid (23.6%).  Figure 1 shows the proportion represented by the antibiotics.

Ofloxacin and the newer fluoroquinolone levofloxacin represented only 20% of the antibiotics prescribed. Intravenous antibiotics were prescribed only to patients diagnosed with pyelonephritis and requiring hospitalization.

### 3.1. Distribution of Bacterial Etiologies

We isolated seven different bacterial species from the 55 urine samples that showed significant bacterial growth. Gram-negative bacteria represented 96.4% of the isolates while only 3.6% were Gram-positive (Table 1).

*Escherichia coli* and* Klebsiella pneumoniae* accounted for the most prevalent bacterial isolates associated with UTI, while* Pseudomonas aeruginosa* and* Staphylococcus saprophyticus* were recovered less often.

*E. coli* was the predominant pathogen among both sexes, followed by* K. pneumoniae* among females. The Gram-positive bacteria,* S. saprophyticus* and* Citrobacter diversus*, were found only in female patients. All isolates found in male patients were also found in female patients except for* Pseudomonas aeruginosa*, which was only found in a male patient.

*E. coli*,* K. pneumoniae*, and* P. mirabilis* were isolated from all age groups. All isolates were detected from the 20–39 years age group.* E. coli* was the predominant isolate in all age groups except for the ≥60 years age group where* E. coli* and* Klebsiella pneumoniae* were recovered in the same proportion. Strata of age gave insufficient evidence for a significant relationship with uropathogens (*p* value = 0.85).

### 3.2. Antibiotic Resistance of Isolates

**Figure 2:**
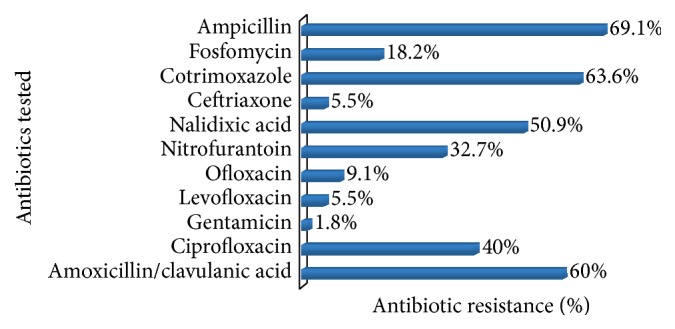
Prevalence of antibiotic resistance (%) among isolates.

Figure 2 shows that most isolates were resistant to ampicillin (69.1%), cotrimoxazole (63.6%), amoxicillin/clavulanic acid (60.0%), and nalidixic acid (50.9%). The prevalence of resistance was low for gentamicin (1.8%), ceftriaxone (5.5%), and fluoroquinolones such as levofloxacin (5.5%) and ofloxacin (9.1%).

Table 2 emphasizes the prevalence of resistance among four predominant bacterial isolates.* E. coli* prevalence was highest for ampicillin (75%) followed by amoxicillin/clavulanic acid (64.3%). Intravenous drugs such as gentamicin and ceftriaxone were the most effective antibiotics against* E. coli*. For oral agents,* E. coli* showed the lowest prevalence of resistance to fluoroquinolones such as levofloxacin (7.1%) and ofloxacin (14.3%); higher prevalence of resistance was noted with ciprofloxacin (21.4%) and cotrimoxazole (60.7%).

Among the bacterial isolates,* K. pneumoniae* had 100% susceptibility to gentamicin, levofloxacin, fosfomycin, and ceftriaxone. The least effective drugs with this isolate were the aminopenicillins.

With* Enterobacter cloacae*, the highest prevalence of resistance was to cotrimoxazole (83.3%), ciprofloxacin (66.7%), and nalidixic acid (60%). This isolate did not exhibit resistance to drugs such as ofloxacin, ceftriaxone, levofloxacin, or gentamicin.

*Proteus mirabilis* isolates were generally very susceptible to our test antibiotics. The highest prevalence of resistance was to cotrimoxazole, ampicillin, and fosfomycin.

**Figure 3:**
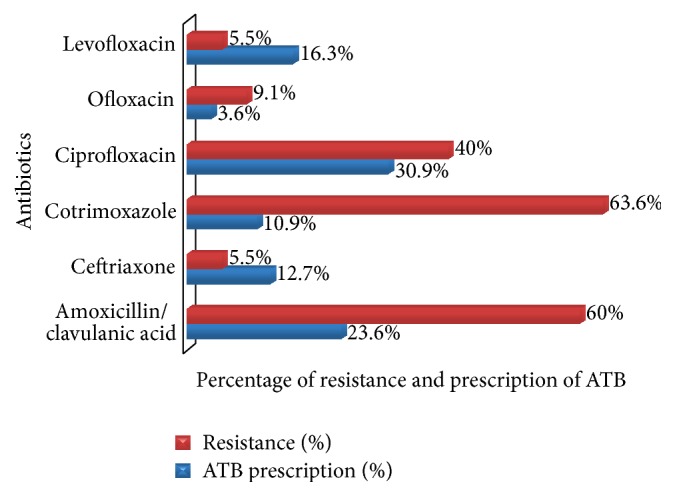
Prevalence of resistance (%) to empirical antibiotics prescribed.

No significant variations in drug resistance were noted among the different age groups or genders. Antimicrobial susceptibility testing showed that 40% of our bacterial isolates were resistant to ciprofloxacin, which was the most empirically prescribed antibiotic. Also, a 60% prevalence of resistance to amoxicillin/clavulanic acid was noted, this being the second most prescribed antibiotic to our set of patients.  Figure 3 shows the prevalence of resistance of bacterial isolates with respect to the antibiotics prescribed to our patients.

## 4. Discussion

In the present study, we sought to determine resistance patterns in prevailing bacterial etiologic agents of CAUTI. We then compared this to prescription habits of local physicians to determine whether changes were necessary. This study provides baseline information that could be used for determining local guidelines for the empirical treatment of CAUTI.

The prevalence (59.8%) of UTI, among symptomatic patients, in this study is similar to the prevalence of 54% recorded in Bamenda by Akoachere et al., 2012, in a similar study [3]. Prevalence rates reported in Cameroon are higher than those reported in other developing countries: 19.3% in Rwanda [8], 10.9% in India [9], and 39.7% in Nigeria [10]. This difference in prevalence could be due to differences in methodology and sample size between these latter studies and our study. The prevalence of UTI was significantly higher in females than in males; these findings agree with earlier similar studies on CAUTIs [3, 10].

Gram-negative organisms (96.4%) were the main cause of UTI in our study, with* E. coli* (50.9%) being the predominant pathogen followed by* K. pneumoniae* (16.4%). This result is similar to findings by Bahadin et al. in Singapore [11]. Also, a study by Gangoue-Piéboji et al., 2004, on 96 urine samples from outpatients at the Yaoundé Central Hospital showed similar results [12]. Our findings were slightly different from those of Akoachere et al. in Cameroon. They found* K. pneumoniae* to be the least isolated uropathogen (1.2%). These variations in etiologic agents could be due to the different bacterial ecology in these different regions. Our study did not show any significant association between age and type of uropathogen isolated or between sex and bacterial etiology. This is similar to the results of other studies [3, 13].

Our study also showed a very low (3.6%) prevalence of Gram-positive pathogens; all of these pathogens were isolated from female patients. This is contrary to other studies [3, 14] which found these pathogens in much larger proportions (>25%) and in both sexes. This could be explained by the fact that we had a smaller sample size compared to those of the above-cited studies.

This study revealed a relatively high prevalence of resistance to most antibiotics tested. Resistance to cotrimoxazole, amoxicillin/clavulanic acid, and ampicillin was particularly alarming. This result is consistent with findings from other studies in Uganda [14] and Buea [3]. Even though high, the prevalence of resistance in Buea was lower than the findings in our study, perhaps due to the population of Yaoundé having more access to antibiotics and hence greater consumption to drive the emergence of resistance. The particularly high prevalence of resistance of our isolates to cotrimoxazole may be explained by additional factors common to our setting, such as the use of cotrimoxazole as prophylaxis among HIV-infected patients and the use of sulfadoxine-pyrimethamine, which shares enzyme targets with cotrimoxazole, for routine malaria prophylaxis during pregnancy.

Although the Infectious Disease Society of America (IDSA) guidelines consider nitrofurantoin and cotrimoxazole for empiric treatment of UTI [15], the prevalence of resistance noted in our study (32.7% and 63.6%, resp.) shows that these drugs may not be appropriate for empiric treatment of UTI in Yaoundé. The most potent drugs were gentamicin, ceftriaxone, ofloxacin, and levofloxacin. This result is similar to the findings of Akoachere et al., 2012 [3], except that the latter did not test levofloxacin.

Our study points out a considerable difference in the prevalence of resistance between ciprofloxacin and the other quinolones tested. This is particularly striking, since the quinolones have the same mechanism of action. When the activities of levofloxacin and ciprofloxacin were compared against bacterial uropathogens, Drago et al. found that resistance to the latter was generally more frequent [16]. Other studies [17, 18] have also noted that ciprofloxacin-resistant bacteria retain susceptibility to newer quinolones. This difference has not been explained.

The antibiotics most frequently prescribed to our patients were ciprofloxacin (30.9%) and amoxicillin/clavulanic acid (23.6%), as was the case in the South African study by Lewis and Perovic [13]. In contrast, cotrimoxazole is most commonly used in the Central African Republic for empirical treatment for CAUTIs [19]. These two antibiotics were among the least potent drugs in our study, suggesting that a change in prescription practice may be necessary. The least prescribed drugs were ceftriaxone, ofloxacin, and levofloxacin. These antibiotics, which exhibited greater potencies than ciprofloxacin and amoxicillin/clavulanic acid, might be a better choice for the empirical treatment of CAUTI, but they have higher cost. Indeed, cost may explain the higher prescription frequency of the less potent drugs. Since the resistance patterns of uropathogens vary considerably between regions and countries [15], a specific treatment recommendation may not be universally suitable. Local data on the prevalence of resistance to antibiotics is likely to be important.

## 5. Study Limitations

We could not verify that* in vitro* resistance to antibiotics prescribed to patients correlated with therapeutic failures. Moreover, limited resources prevented us from obtaining a larger sample that might better represent the population of Cameroon.

## 6. Conclusion

The present study reveals a familiar pattern with respect to the species of uropathogens involved in CAUTIs, and it showed considerable bacterial resistance to common empirically prescribed antibiotics. The work suggests that newer fluoroquinolones are better for the empirical treatment of CAUTIs.

## Figures and Tables

**Table 1:** Frequency of bacterial isolates.

Bacterial isolates	Frequency (%)	Gram reaction
*Escherichia coli*	28 (50.9%)	Gram-negative *N* = 53 (96.4%)
*Proteus mirabilis*	5 (9.1%)	
*Klebsiella pneumoniae*	9 (16.4%)	
*Enterobacter cloacae*	6 (10.9%)	
*Citrobacter diversus*	4 (7.3%)	
*Pseudomonas aeruginosa*	1 (1.8%)	

*Staphylococcus saprophyticus*	2 (3.6%)	Gram-positive *N* = 2 (3.6%)

Total bacterial isolates	55 (100%)	

**Table 2:** Prevalence of antibiotic resistance among the predominant isolates.

Antibiotics	*E. coli* (%) *N* = 28	*K. pneu* (%) *N* = 9	*E. cloacae* (%) *N* = 6	*P. mirabilis* (%) *N* = 5
Amoxicillin/clav. acid	64.3	100	50	20
Ampicillin	75	100	33.3	40
Ceftriaxone	7.1	11.1	0	0
Ciprofloxacin	21.4	55.6	66.7	20
Cotrimoxazole	60.7	44.4	83.3	60
Fosfomycin	21.4	0	16.7	40
Gentamicin	3.4	0	0	0
Levofloxacin	7.1	0	0	0
Nalidixic acid	50	55.6	60	20
Nitrofurantoin	25	55.6	40	20
Ofloxacin	14.3	0	0	20

## Abbreviations

IDSA: Infectious Disease Society of America
CAUTI: Community-acquired urinary tract infection
YUHC: Yaoundé University Hospital Centre
YCH: Yaoundé Central Hospital
CFU: Colony forming units
WBC: White blood cell
FMBS: Faculty of Medicine and Biomedical Sciences.
